# Hydrous icaritin nanorods with excellent stability improves the *in vitro* and *in vivo* activity against breast cancer

**DOI:** 10.1080/10717544.2020.1716877

**Published:** 2020-01-31

**Authors:** Yian Wang, Tiantian Huang, Haowen Li, Jingxin Fu, Hui Ao, Likang Lu, Meihua Han, Yifei Guo, Feng Yue, Xiangtao Wang

**Affiliations:** a Institute of Medicinal Plant Development, Chinese Academy of Medical Sciences, Peking Union Medical College, Beijing, PR China;; b School of Pharmacy, Henan University of Traditional Chinese Medicine, Zhengzhou, PR China;; c Guangdong Jiabo Pharmaceutical Co. Ltd., Guangdong, PR China

**Keywords:** Flavonoids, hydrous icaritin, nanorods, anti-breast cancer

## Abstract

Due to their various biological activities that are beneficial to human health and antitumor effect, flavonoid compounds have attracted much attention in recent years. Hydrous icaritin (HICT) was such a flavonoid that can inhibit the growth of breast cancer and cancer stem cells. In order to overcome the insolubility problem, HICT was fabricated into nanorods (NRs) through anti-solvent precipitation in this paper using D-α tocopherol acid polyethylene glycol succinate and sodium oleate as a co-stabilizer meanwhile using the mixture of ethanol and acetone (1:2, v/v) as the organic solvent. The obtained HICT NRs showed an average particle size 222.0 nm with a small polydispersity index value of 0.124 and a high zeta potential of – 49.5 mV. HICT NRs could maintain similar particle size in various physiological medium and could be directly lyophilized without the addition of any cytoprotectants and then reconstituted into a colloidal system of similar size. The resultant HICT NRs had a high drug loading content of 55.6% and released HICT in a steady and constant pattern. MTT assay indicated NRs enhanced HICT’s antitumor activity to ninefold against MCF-7 breast carcinoma cells. *In vivo* studies demonstrated oral administration free HICT had almost no tumor inhibitory effect while HICT NRs showed a tumor inhibition rate of 47.8%. When intravenously injected, HICT NRs displayed similar therapeutic efficacy to paclitaxel injections (70.4% vs. 74.5%, TIR). This may be partly due to the high accumulation of the injected HICT NRs in tumor ranking only second to that in the liver but much higher than in other organs. These results demonstrated that HICT NRs could be a promising antitumor agent for the treatment of breast cancer in clinic.

## Introduction

The burden of cancer is increasing worldwide due to the aging of population and the increasing of unhealthy lifestyles (Torre et al., 2015). Breast cancer has been the highest incidence of cancer among women (Torre et al., 2015; Chen et al., 2018). However, the current therapeutic methods, including hormone therapy, radiotherapy, surgery, and chemotherapy, are not satisfied due to the accompanied side effect and multiple drug resistance (Peng et al., 2009; Akram et al., 2017; Chatterjee & Bivona, 2019), which will in return aggravate the patient’s depression (Rao et al., 2019), the most widely occurred symptom among the breast cancer patients (Zainal et al., 2013; Shah et al., 2014; Stanton & Bower, 2015).

Natural products prove to be one of the sources of antitumor drugs, such as paclitaxel and hydroxylcamptothecin. Flavonoids are a major class of polyphenols naturally abundant in various fruits, vegetables, tea, and plants. Due to their various biological activities beneficial to human health and antitumor effect, flavonoid compounds are attracting much attention in recent years (Perez-Vizcaino & Fraga, 2018; Rauf et al., 2018).

Hydrous icaritin (HICT) is just such a flavonoid compound that can not only effectively inhibit the growth of breast cancer cells (Pan et al., 2007; Pan et al., 2010; Guo et al., 2011; Lai et al., 2013; Wang et al., 2017; Zhang et al., 2018) and cancer stem cells (Guo et al., 2011), but also relieve depression (Pan et al., 2007; Pan et al., 2010). However, like most flavonoids, HICT is water-insoluble, thus difficult to be effectively delivered *in vivo*, and meanwhile results into low bioavailability and limited effectiveness (Lewin et al., 2013; Guo et al., 2017). Encapsulation of hydrophobic drugs into nanoparticles, micelles or liposomes can well resolve the insolubility problem of this kind of drugs and achieve passive targeting through the enhanced permeability and retention (EPR) effect when intravenously administrated (Hashizume et al., 2000; Torchilin, 2011; Maeda et al., 2013; Prabhakar et al., 2013), and is thus suitable for hydrophobic antitumor agents. In this paper, HICT was successfully fabricated into nanorods (NRs) with good stability and high drug loading content (DLC). As far as we know, it is the first report on HICT NRs and the first report on NRs for flavonoid compounds.

More and more investigations have proved that the shape has a fundamental effect on the *in vivo* fate of nanoparticles through their interaction with the plasma components, immune system and the various physiological barriers and so on. NRs show longer blood circulation time, higher cellular uptake and higher tumor accumulation than disc and spherical nanoparticles (Toy et al., 2014; Banerjee et al., 2016; Yang et al., 2016). Probably due to these reasons, the obtained HICT NRs showed significantly increased the *in vitro* antitumor effect in comparison with free HICT, much significantly enhanced tumor inhibition rate in contrast with free HICT when oral administrated and a similar *in vivo* antitumor therapeutic efficacy to that of paclitaxel (PTX) injections. This demonstrated that HICT NRs are promising to be an effective antitumor drug in the future.

## Materials and methods

### Materials

Sodium oleate (SO) was obtained from Bio-Ruler Company. HICT was supplied by Nanjing Dasfbio Co. Ltd (Nanjing, China). D-α tocopherol acid polyethylene glycol succinate (TPGS), PCL_2k_-mPEG_2k_ and PLA_2k_-mPEG_2k_ were supplied by Xi’an Healthful Biotechnology Co. Ltd (Xi’an, China). Soybean phosphatidylcholine (SPC) was purchased from Jinan Dai gang Biomaterial Co. Ltd (Jinan, China). Bovine Serum Albumin (BSA) was obtained from Beijing Bio topped Science & Technology Co, Ltd. Dir iodide [1-1-dioctadecyl-3,3,3,3-tetramethlindotricarboc-yanine iodide] (Dir) was obtained from AAT BioQuest (Sunnyvale, CA, USA). PTX injection was purchased from Beijing Union Pharmaceutical Factory (Beijing, China). 3-(4,5-dimethylthiazol-2-yl)-2,5-diphenyltetrazolium bromide (MTT) were purchased from Sigma-Aldrich (St. Louis, MO, USA). Acetonitrile [high-performance liquid chromatography (HPLC) grade] was from Fisher Scientific (Pittsburgh, PA, USA). Deionized water used in the experiments.

### Animals and cell lines

The MCF-7 (breast carcinoma) cell line was supplied by China infrastructure of cell line resource. The cells were cultured in DMEM medium with 10% fetal calf serum (Thermo Fisher Scientific), streptomycin (100 U/mL), and penicillin (100 U/mL) at 37 °C with 5% CO_2_ in cell incubator (Sanyo, Osaka, Japan). Female NU/NU nude mice (20 ± 2 g, 6–8 weeks old) were obtained from Vital River Laboratory Animal Technology Co., Ltd. (Beijing, China). The animals were acclimated under 12 h light–dark cycle environment with relative humidity of 70 ± 5% at 25 °C for one week. All animal experiments are conducted according to guidelines for Ethical and Regulatory for Animal Experiments Stipulated by the Institute of Medicinal Plant Development (IMPLAD).

### Preparation of HICT NRs

#### Screening for suitable stabilizer

HICT NRs were fabricated by the antisolvent precipitation method. Preliminary test showed HICT had much higher solubility in dimethyl sulfoxide (DMSO) (>50 mg/mL) than in other solvents such as ethanol, acetone, and methanol, so DMSO was firstly selected as the organic solvent. Briefly, 5 mg of HICT powder and 5 mg of stabilizer were co-dissolved in 0.3 mL of DMSO according to a fixed HICT/stabilizer feeding ratio (1:1, weight ratio), then slowly instilled into 5 mL of deionized water under ultrasonic conditions (250 W, 25 °C), followed by centrifugation at 13000 r/min for 10 min. The sediment was resuspended in 5 mL of deionized water, sonicated for 10 min, and then homogenized (25 °C, 1500 bar) for 10 cycles to obtain HICT NRs. When mPEG_2000-_PCL_2000_, mPEG_2000_-PLA_2000_, Oleic acid, TPGS and SPC were used as a stabilizer, respectively, they were dissolved together with HICT in DMSO; while BSA was dissolved in the deionized water when used as a stabilizer.

#### Improvement of HICT nanorods stability by alteration of organic solvent

5 mg of TPGS and 5 mg of HICT were dissolved in 0.5 mL of acetone and ethanol (2:1, v/v), then instilled into 5 mL of deionized water under ultrasonic conditions (250 W, 25 °C), followed by rotary evaporation at 40 °C to remove the organic solvents, then the mixture was homogenized (25 °C, 1500 bar) for 10 cycles. Then the particle size of the resultant HICT NRs was monitored during their incubation in various physiological medium at 37 °C.

#### The optimization of HICT NR preparation

Different HICT/TPGS feeding ratio (2:1, 3:1, 4:1) was tried according to the method described in the method ‘Improvement of HICT nanorod’s stability by alteration of organic solvent’ and their particle size and polydispersity index (PDI) values were measured to select the optimal feeding ratio.

In order to reduce the intestinal metabolism of HICT when orally administrated, SO was added in the formulation of HICT nanosuspension. Specifically, 9 mg of HICT and 3 mg of TPGS were co-dissolved in 0.9 mL of acetone and ethanol (2:1, v/v), then instilled into 9 mL of deionized water containing 3 mg of SO, followed by rotary evaporation at 40 °C to remove the organic solvents, then the mixture was homogenized (25 °C, 1500 bar) for 10 cycles to obtain HICT NRs.

### Physicochemical characterization of HICT NRs

#### Dynamic light scattering measurement

The mean particle diameter, PDI, and zeta potential of HICT NRs were measured by a dynamic light scattering method (Zetasizer Nano ZS, Malvern Instruments, UK) at 25 °C. Each sample was measured three times and all data were expressed in the form of mean ± standard deviation (SD).

#### Morphology of HICT NRs

The morphological characterization of HICT NRs was performed by a JEM-1400 transmission electron microscope (JEOL, Tokyo, Japan). A drop of particle dispersion was dropped onto the surface of the 300-mesh copper mesh, dyed with 2% (w/v) uranyl acetate, and then the morphology of HICT NRs was observed under the electron microscope.

#### The stability of HICT NRs in various physiological medium

HICT NRs were, respectively, mixed with 1.8% NaCl, 10% glucose, 2 × PBS (1:1, v/v), or mixed with plasma, simulated gastric fluid (1% pepsin in 1 mol/L diluted HCl), and simulated intestinal fluid (1% pancreatin in pH 6.8 PBS, 0.01 M) (1:4, v/v) and incubated at 37 °C. Particle size and PDI value of the sample were measured at specific time intervals. The above experiments were conducted in triplicates.

#### Lyophilization of HICT NRs

20 mL of HICT NRs solution was taken in a beaker and covered with lens paper frozen for 6 h under –80 °C freezer (MELING DW-HW50, China) and lyophilized for 12 h in a vacuum freeze dryer (FD, Christ-Alpha 2-4 LD plus, Germany) under 0.12 mbar, –40 °C. The freeze-dried powder was dispersed in 20 mL deionized water and the particle size, PDI, and zeta potential were measured as described in the method of ‘dynamic light scattering measurement.’

#### Chromatographic condition of HICT

The concentration of HICT was measured using HPLC system (DIONEX Ultimate 3000, USA) (HPLC, DIONEX Ultimate 3000, Germering, Germany). A column C_18_ (4.6 mm × 250 mm, 5 μm; Dr. Maisch GmbH, Germany) was used at 25 °C for chromatographic separation. The mobile phase was constituted of acetonitrile and 0.1% acetate water (68:32, v/v) with a flow rate of 0.6 mL/min. The detection wavelength UV was 270 nm.

#### Drug loading content of HICT NRs

To determine DLC of HICT NRs, the lyophilized HICT NRs were weighed and dissolved in methanol. The concentration of HICT was determined using HPLC system. The DLC of HICT NRs is expressed as follows:

(1) $$\mathrm{DLC}\left(\%\right)=\frac{V\cdot C}{W}\times 100\%,$$

where *V* is the volume of methanol, *C* is the concentration of HICT, and *W* is the weight of lyophilized powder of HICT NRs.

#### *In vitro* drug release behavior

The release behavior of HICT NRs (300 μg/mL, 10 mL) was performed in triplicate in Float-A-Lyzer dialysis cassettes (MWCO: 20KD, Spectrum Labs, Rancho Dominguez, CA, USA). Then the dialysis cassettes were suspended in 2.0 L PBS (PH 7.4 ± 0.1, 2.0 L) containing 0.5% (w/v) tween 80 to assure the sink conditions at 37 °C with constant stirring (100 rpm). 200 μL of internal dialysate were withdrawn at fixed time intervals, the same volume of fresh release medium was replenished and the external release medium was replaced every 24 h. HICT concentration in the dialysis cassettes was determined by HPLC. The cumulative release of drug was calculated by reduction of HICT NRs inside the dialysis cassettes.

#### Differential scanning calorimetry (DSC) characterization

DSC thermal profiles of the powder samples were tested through a differential scanning calorimeter (Q200, TA Instruments, New Castle, DE). Samples of approximately 10 mg were placed in standard aluminum pans, sealed with a lid and measured from 0 to 300 °C at a scanning rate of 10 °C/min under nitrogen atmosphere.

#### X-ray diffraction (XRD) measurements

An X-ray diffractometer (DX-2700, China) was applied to conduct X-ray powder diffraction with a generator set at 40 kV and 100 mA. Samples were scanned over an angular range of 3–80° of 2θ, with a step size of 0.02° and a count time of three per step. Samples were rotated at 30 rpm during the analyses.

### The pharmacological evaluation

#### *In vitro* cytotoxicity assay

The cytotoxicity of HICT and HICT NRs were determined by MTT assay. First, the MCF-7 cells in the logarithmic growth phase were cultured in 96-well plates (8000 cell/well), and cultured in 37 °C, 5% CO_2_ environment for 24 h. Then HICT NR and HICT DMSO solution (dissolved in DMSO, final concentration of DMSO ≤ 0.1%) was diluted into different concentrations by medium and added to each well. After 48 h of continuous culture, 20 μL MTT solution (5 mg/mL in PBS) was added to each well and incubated for 4 h. Then the supernatant solution was decanted, 150 μL of DMSO was added dropwise to each well to dissolve the bottom precipitate, and the optical density (OD) value was measured at a maximum absorption wavelength of 570 nm by ELISA plate reader (Biotech, Winooski, VT, USA). The cell inhibition rate of HICT NRs and HICT DMSO solution is calculated by the following formula:

(2) $$\mathrm{Inhibition}\;\mathrm{rate}\left(\%\right)=\left(1-\frac{{OD}_{e}}{{OD}_{C}}\right)\times 100\%,$$

where OD_e_ is the mean OD of experimental group and OD_c_ is the mean OD of control group. The half-maximal inhibitory concentration (IC_50_) of HICT NRs was calculated from GraphPad Prism software, version 5.0 (GraphPad Software, La Jolla, CA, USA), by the sigmoidal dose–response variable curve-fitting method.

#### *In vivo* antitumor efficacy and biodistribution

*In vivo* antitumor efficacy of HICT NRs was investigated using MCF-7 NU/NU tumor-bearing nude mice. Female NU/NU nude mice were inoculated with MCF-7 cells (4.0 × 10^7^/mL, 0.2 mL/mouse) at the right forelimb. When the tumor volume reached 100 mm^3^, the nude mice were randomly divided into five groups (six mice in each group). Three groups of mice were, respectively, administered normal saline (negative control, 0.2 mL/mouse), PTX injection (positive control, 8 mg/kg) and HICT-NRs (35 mg/kg) via tail vein every other day for 14 days. The other two groups of mice were, respectively, given daily gavage of HICT-NRs (35 mg/kg) and free HICT (HICT, TPGS and SO were composed of suspension dispersed in water at a mass ratio of 3:1:1) for 14 days. The weight of mice and length (a) and width (b) of the mice tumor were measured every other day. The tumor volume was calculated by the formula: *V* = (a·b^2^)/2. At the end of the experiment (12 h after the last dose), the mice were sacrificed by spine. The tumor, liver and spleen were dissected and weighed, respectively.

The tumor inhibition rate and liver and spleen index are calculated according to the following formulas..

The tumor inhibition rate (TIR):

(3) $$\mathrm{TIP}\left(\%\right)=\left(1-\frac{W_{e}}{W_{n}}\right)\times 100\%,$$

where *W*_e_ is the mean tumor weight of experimental group and *W*_n_ is the mean tumor weight of negative control group.

Liver index rate (LIR):

(4) $$\mathrm{LIR}=\sum_{i=1}^{6}\frac{W_{Li}}{W_{mi}}÷6,$$

where *W_Li_*is the liver weight of experimental group and *W_mi_*is the nude mice weight of group.

Splenic index rate (SIR):

(5) $$\mathrm{SIR}=\sum_{i=1}^{6}\frac{W_{Si}}{W_{mi}}÷6,$$

where *W_Si_*is the spleen weight of experimental group and *W_mi_*is the nude mice weight of group.

In order to know the biodistribution and tumor-targetability of HICT NR, Dir-labeled HICT NRs were prepared according to the finally determined method just by incorporating a bit of Dir (1:40, Dir: HICT, weight ratio) into the organic phase, and then the resultant Dir-labeled HICT NRs were intravenously injected through the tail vein of mice for the last dose. At the end of the experiment, the major organs such as heart, liver, spleen, lung, kidney and brain together with tumor were put to measure the fluorescence using the IVIS Living Image software, version 4.4 (Caliper Life Sciences, Hopkinton, MA, USA).

### Data analysis

Statistical analysis of the experimental data was performed using Statistical Package for the Social Sciences software, and IC_50_ values were calculated by GraphPad Prism software, version 6.01 (GraphPad Software, La Jolla, CA, USA). *In vitro* and *in vivo* results were analyzed by *t-*test and one-way analysis of variance. The value of *p* < .05 was considered statistically significant.

## Results and discussion

### Preparation and optimization of HICT NRs

#### Screening for suitable stabilizer

Preliminary test showed HICT had much higher solubility in DMSO (> 50 mg/mL) than in ethanol, acetone, and methanol, so DMSO was firstly selected as the organic solvent to prepare HICT NRs. As shown in Table 1, each of the listed stabilizers could alone help to fabricate HICT into NRs with small particle size (<230 nm) and narrow particle distribution (PDI < 0.2). Although the obtained HICT NRs basically maintained their particle size in normal saline (Table 1), they were all unstable in PBS, 5% glucose, simulated gastric fluid and simulated intestinal fluid with significantly increased particles size and aggregation (Supplementary Tables S1–S6), among which TPGS showed the best performance (Supplementary Table S4). Only when the NRs are stably present in the body fluids, the advantages of NRs can be achieved. So, it is necessary to tune the preparation of HICT NRs.

**Table 1.** The particle size, PDI, and zeta potential of HICT nanorods before and after 10 h of incubation in normal saline.

Carriers	Initial			In normal saline for 10 h		
	Size (nm)	PDI	Zeta (mV)	Size (nm)	PDI	Zeta (mV)
PCL_2k_**-**mPEG_2k_	214.3 ± 3.0	0.06 ± 0.04	–13.9 ± 0.3	263.2 ± 3.0	0.23 ± 0.04	–8.43 ± 0.3
PLA_2k_**-**mPEG_2k_	226.9 ± 2.8	0.19 ± 0.06	–19.0 ± 0.4	265.3 ± 4.6	0.28 ± 0.02	–6.15 ± 0.1
Oleic acid	196.0 ± 5.8	0.19 ± 0.04	–23.4 ± 0.1	279.4 ± 3.4	0.32 ± 0.05	–70.5 ± 1.0
TPGS	217.1 ± 3.4	0.18 ± 0.03	–19.5 ± 0.5	225.6 ± 6.7	0.20 ± 0.02	–20.5 ± 0.6
SPC	216.9 ± 1.0	0.18 ± 0.02	–13.9 ± 1.2	445.2 ± 37	0.46 ± 0.06	–12.9 ± 0.2
BSA	184.2 ± 2.2	0.18 ± 0.05	–7.41 ± 1.5	392 ± 11	0.38 ± 0.04	–10.1 ± 0.2

SD: standard deviation.

#### Improvement of HICT NR stability in physiological medium

There are many factors influencing the self-assembly of hydrophobic molecules and stabilizer during the antisolvent precipitation process, such as the type of stabilizer, the type of organic solvent, the feeding ratio of drug and stabilizer, the removal rate of the organic solvent and so on.

Among all the tested mixing systems composed of ethanol, acetone, and methanol, the mixture of ethanol and acetone (1:2, v/v) showed the best solubility for HICT (about 8.5 mg/mL), and thus was tried as the good solvent for the preparation of HICT NRs through antisolvent precipitation. Fortunately, as shown in Table 2, HICT NRs obtained in this way proved to be able to stably exist in various physiological medium with similar particle size and acceptable PDI values after 10 h incubation, and no aggregation or abnormal phenomena was observed. Thus, the mixture of ethanol and acetone (1:2, v/v) was chosen as the good solvent for the preparation of HICT NRs in the subsequent study.

**Table 2.** Particle size, PDI, and zeta potential of HICT NRs before and after 10 h of incubation in various physiological medium (5% Glu, 0.9% NaCl, PBS, simulated gastrointestinal fluid).

Medium	Size (nm)	PDI	Zeta (mV)
Initial	265.3 ± 7.4	0.195 ± 0.01	–25.3 ± 0.1
PBS	294.9 ± 1.8	0.21 ± 0.02	–1.4 ± 0.3
5% Glu	295.1 ± 4.0	0.27 ± 0.01	–14.1 ± 0.9
0.9% NaCl	288.0 ± 1.0	0.26 ± 0.03	–2.3 ± 0.6
Gastric fluid	316.8 ± 5.6	0.34 ± 0.01	6.6 ± 0.4
Intestinal fluid	325.7 ± 3.4	0.25 ± 0.04	–2.2 ± 0.5

SD: standard deviation.

**Figure 1.**
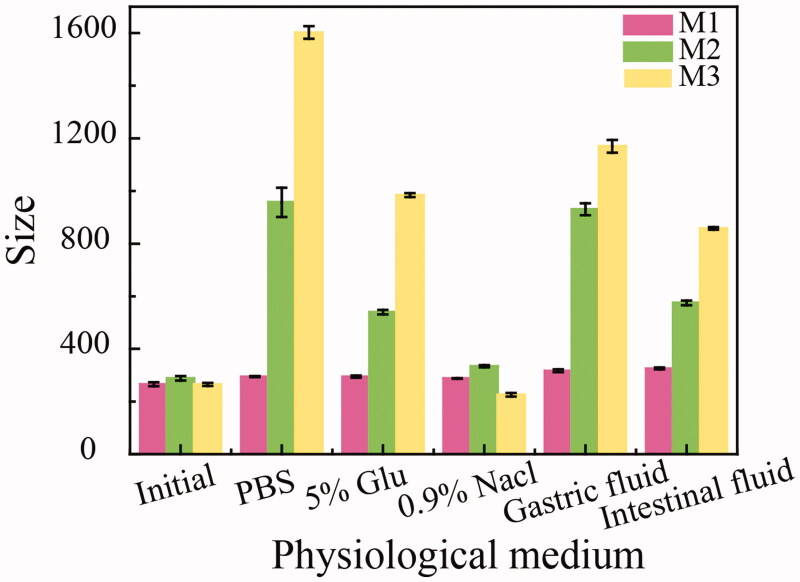
The particle size of HICT NRs prepared by different methods after 10 h of incubation in various physiological medium (normal saline, PBS, 5% glucose, simulated gastric juice, simulated intestinal juice) at 37 °C. M1: Ethanol and acetone were used as organic solvents and removed by rotary evaporation after formation of HICT NRs. M2: Ethanol and acetone were used as organic solvents and removed by centrifugation after formation of HICT NRs. M3: DMSO was used as organic solvents and removed by centrifugation after formation of HICT NRs. Data represented as the mean ± SD.

It was speculated that the instability of the HICT NRs prepared using DMSO as the good solvent was partly ascribed to the specific self-assemble mode of HICT and stabilizer when the DMSO solution was diluted with deionized water, and partly ascribed to the much less of reservation of the feeding stabilizer, most of which remained in the supernatant during the centrifugation. In order to explore the reason behind, three different HICT NRs were prepared using TPGS as a stabilizer with a HICT/TPGS feeding ratio of 1:1, all through antisolvent precipitation method. The first one used the mixture of ethanol and acetone (1:2, v/v) as the good solvent to prepare HICT NRs (named as M1) and then removed the solvent by vacuum rotary evaporation; the second one used the mixture of ethanol and acetone (1:2, v/v) as the good solvent (named as M2) and then removed the solvent by centrifugation; the third one used DMSO as the good solvent (named as M3) and then removed DMSO by centrifugation. The particle size of the three HICT NRs after incubation in the physiological medium at 37 °C for 10 h was examined. As shown in Figure 1, M2 NRs showed significantly increased particle size in PBS, simulated gastric and simulated intestinal fluids in contrast to M1, indicating that removal of partial stabilizer that retained in the supernatant reduced the stability of the M2 NRs. Removal of the organic solvent by vacuum rotatory evaporation is a relatively slow process, thus probably allow M1 NRs to ‘repair’ their structural flaws during the solvent evaporation. However, during the centrifugation, the self-assembly NRs fail to ‘repair’ their structural flaws due to an abrupt loss of the organic solvent and the co-existed stabilizer that was abundant in the supernatant. The all above factors may result in the less stability of M2 and M3 HICT NRs in comparison with M1 NRs. In addition, the self-assembly of HICT and TPGS in the ethanol–acetone–water system may also differ from that in the DMSO–water system.

**Figure 2.**
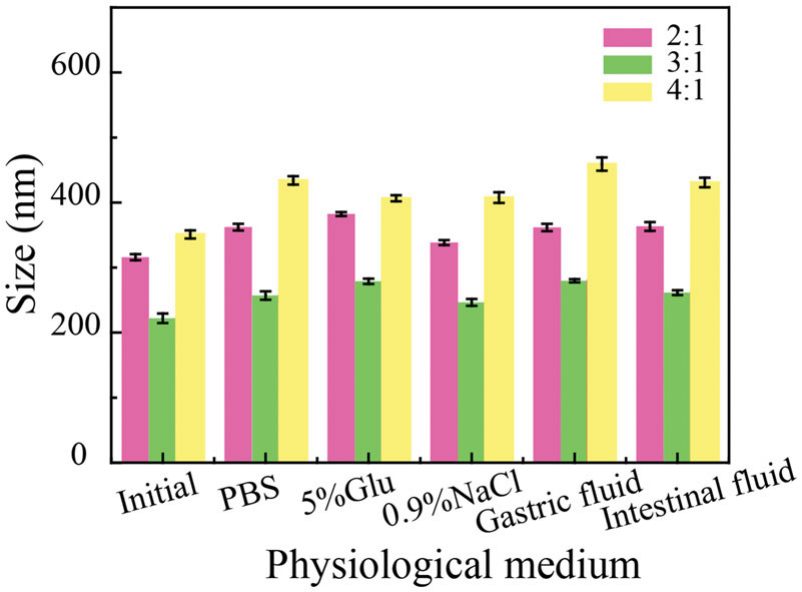
The particle size of HICT NRs after 10 h of incubation in various physiological medium (normal saline, PBS, 5% glucose, simulated gastric juice, simulated intestinal juice) at 37 °C. HICT NRs were prepared at different feeding ratios of 2:1, 3:1 and 4:1, respectively.

#### Formulation optimization and lyophilization

Small particle size, good stability in the physiological medium and high DLC are all that we want for HICT NRs. As seen in Figure 2, it was evident the HICT/TPGS feeding ratio of 3:1 well balanced the small particle size and presentable DLC and meanwhile achieved good stability in various physiological medium. So, the HICT/TPGS feeding ratio of 3:1 was chosen to prepare HICT NRs in the subsequent study.

It has been reported that most flavonoids are subject to phase II metabolism and the widely occurred phase II metabolism becomes one of the main reasons for the low oral bioavailability for flavonoids (Dong et al., 2017). Thus, SO, a strong phase II metabolism inhibitor (He et al., 2018), was added in the formulation of HICT NRs with the aim of improving the oral bioavailability of HICT. As seen in Table 3, the incorporation of SO not only has little effect on the particle size and PDI values of HICT NRs, but also increased their zeta potential, which is beneficial to the stability of NRs. Unexpectedly, through the incorporation of SO, the resultant HICT NRs could be lyophilized without any cryoprotectant and then be reconstituted into a clear colloidal system with the similar particle size just by re-suspending into water followed by gentle shaking (Table 3).

**Table 3.** The particle size, PDI, and zeta potential of HICT NRs before and after lyophilization and reconstitution.

Formulation	Size (nm)	PDI	Zeta (mV)
HICT/TPGS NRs			
Before lyophilization	209.8 ± 4.7	0.151 ± 0.047	–34.5 ± 1.5
After lyophilization	570.0 ± 8.9	0.376 ± 0.022	–21.0 ± 0.91
HICT/TPGS/SO NRs			
Before lyophilization	222.0 ± 7.4	0.124 ± 0.009	–49.5 ± 1.2
After lyophilization	250.2 ± 7.2	0.219 ± 0.009	–44.5 ± 3.37

SD: standard deviation.

So far, the final formulation of HICT/TPGS/SO (3:1:1) was set with the specific preparation method as follows: HICT and TPGS were co-dissolved in acetone and ethanol (2:1, v/v), then the organic solution was instilled into 10 volume of deionized water containing SO, followed by rotary evaporation at 40 °C to remove the organic solvents, and then homogenization (25 °C, 1500 bar) for 10 cycles to obtain HICT NRs.

#### Characterization of HICT NRs and stability of physiological medium

**Figure 3.**
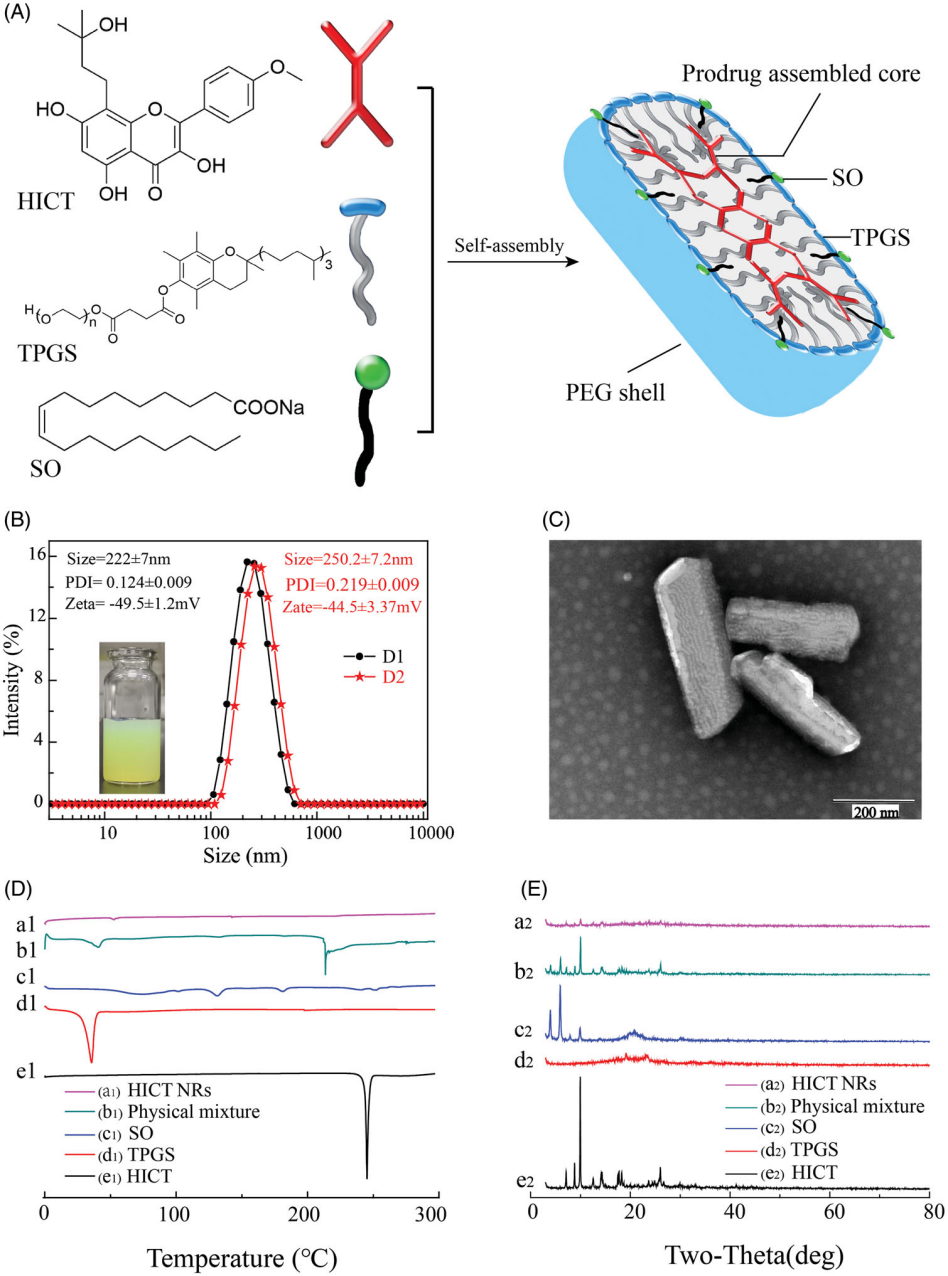
Characterization of HICT NRs. (A) The illustration of the formation of HICT NRs. (B) The photo and particle size of HICT NRs (D1: HICT NRs before lyophilization; D2: HICT NRs after lyophilization and then being suspended in water). (C) TEM image of HICT NRs (scale bar was 200 nm). (D) DSC patterns of HICT NRs, HICT bulk powder, TPGS, SO, the physical mixture of HICT powder, SO and TPGS. (E) XRD patterns of HICT NRs, HICT bulk powder, TPGS, SO, physical mixture of HICT bulk powder, SO, and TPGS.

The self-assembly of HICT, TPGS and SO into NRs was illustrated in Figure 3(A). The resultant HICT NRs had an average particle size of 222.0 ± 7.4 nm with a small PDI of 0.124 ± 0.035, and a high zeta potential of (–44.5 ± 3.37) mV (Figure 3(B)). TEM photography displayed that HICT NRs were NRs in shape (Figure 3(C)), which was very seldom seen for flavonoid nanoparticles. It was determined by HPLC assay that the actual DLC of HICT NRs was 55.6%, a litter lower than the theoretic value of 60%.

In the DSC diagram, bulk HICT powders showed sharp and strong diffraction peaks at 245.28 °C (Figure 3(D)-e1) corresponding to the melting point of HICT, indicating its crystal characteristics. However, this peak was shifted in the physical mixture of HICT, TPGS and SO (Figure 3(D)-b1), and disappeared in HICT NRs (Figure 3(D)-a1), indicating the alteration of the original crystalline form of HICT and the fact that HICT are easy to interact with TOGS and SO during the heating process. And the total disappearance of this peak in the DSC diagram of HICT NRs (Figure 3(D)-a1) suggested HICT may be in the amorphous form in HICT NRs.

In the XRD spectrum of the physical mixture (Figure 3(E)-b2), there is a lot of sharp diffraction peaks corresponding to those of HICT bulk powder (Figure 3(E)-e2). However, in the XRD spectrum of HICT NRs (Figure 3(E)-a2), no significant diffraction peaks of HICT could be observed, indicating the transform of HICT crystalline into amorphous form during the preparation of HICT NRs, which was in accordance with the result of DSC. High energy state of HICT (amorphous form) in HICT NRs meant the probably relatively fast drug release *in vitro* and *in vivo*.

#### The stability of HICT NRs in various physiological medium and drug release *in vitro*

**Figure 4.**
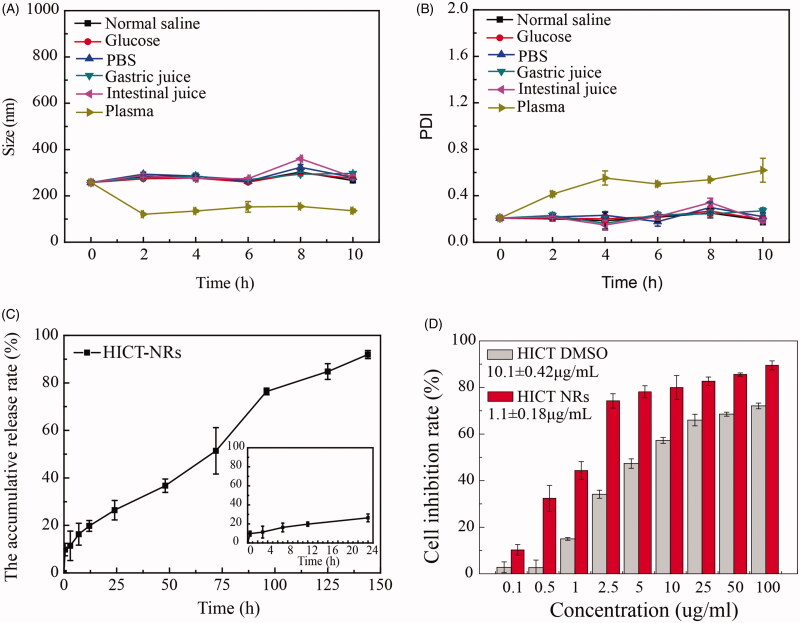
The stability in various physiological medium, *in vitro* drug release and cytotoxicity of HICT NRs. (A) For the average particle size of HICT NRs in various physiological medium. (B) For the PDI of HICT NRs in various physiological medium. (C) For the cumulative drug release of HICT NRs in PBS (pH 7.4 ± 0.1) containing 0.5% (w/v) tween 80 at 37 °C. (D) For the cell growth inhibition of HICT NRs and free HICT against MCF-7 cell line after 48 h incubation, with the IC_50_ being 1.1 ± 0.18 μg/mL for HICT NRs and 10.1 ± 0.42 μg/mL for free HICT (*p* < .01). Data represented as mean ± SD.

In order to achieve effective *in vivo* drug delivery, HICT NRs must be stable in various physiological medium. As seen in Figure 4(A, B), the obtained HICT NRs well maintained their original particle size with similar PDI values in normal saline, PBS, 5% glucose, plasma, simulated gastric and intestinal fluids with no turbidity or aggregation at all. The only difference was that HICT NRs in plasma was smaller than that in deionized water. This was because that blank plasma itself contained some tiny nanoparticles (probably composed of serum proteins) and nanovesicles (e.g. exosomes), and their presence in the mixture led to the relatively smaller particle size and relatively higher PDI value as a system when HICT NRs mixed with plasma. Similar phenomena have been observed and reported (Allen et al., 1999; Yang & Alexandridis, 2000).

The *in vitro* release of HICT NRs was investigated in PBS solution (pH = 7.4) containing 0.5% Tween 80. As shown in Figure 4(C), HICT NRs displayed a steady and constant drug release pattern and the cumulative release reached about 90% in 140 h. The sustained release could help avoid drug leakage in circulation and was beneficial for drug to be effectively delivered into tumor through EPR effect.

#### *In vitro* cytotoxicity experiment

MTT assay was used to examine the *in vitro* inhibitory effect of HICT NRs against breast cancer MCF-7 cell using free HICT as a control. As shown in Figure 4(D), in the concentration range of 0.5–100 ug/mL, both HICT NRs and free HICT displayed a good dose-dependent inhibition against the growth of MCF-7 cells; however, HICT NRs showed stronger inhibitory effect than free HICT at each concentration. It was calculated the IC_50_ of HICT NRs was 1.1 µg/mL, much lower than that of free HICT (10.1 µg/mL, *p* < .01). This demonstrated that after fabrication in NRs, the *in vitro* antitumor activity of HICT was enhanced to more than ninefold, which may be due to that the unspecific interaction between NRs and cells promoted cellular uptake of HICT nanoparticles and the shape of NR further facilitated this process.

#### The *in vivo* antitumor treatment and biodistribution

**Figure 5.**
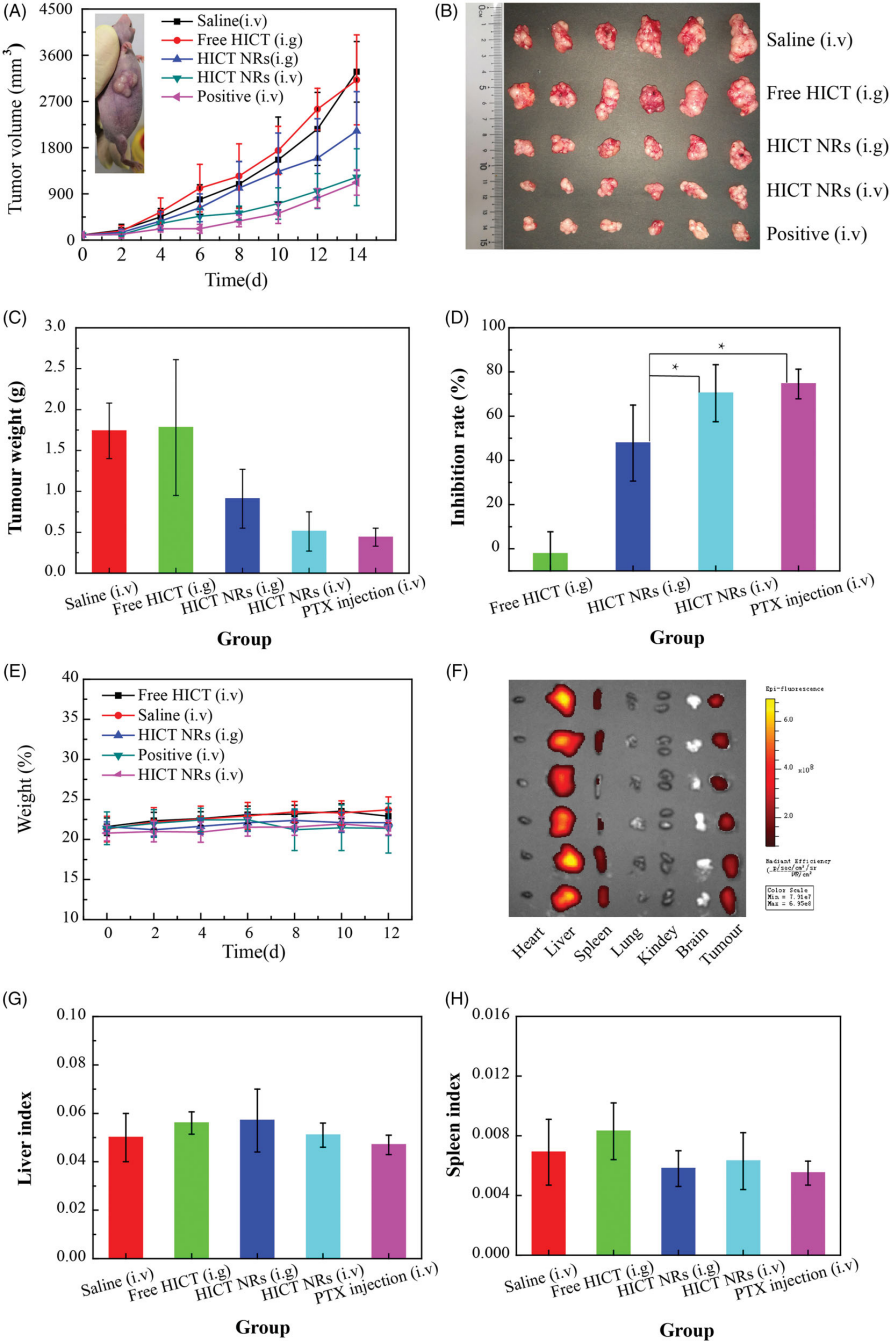
*The in vivo* antitumor study of HICT NRs on MCF-7 tumor-bearing nude mice model at the equivalent HICT dose of 35 mg/kg using 8 mg/kg of paclitaxel injections (dosed every other day) as a positive control. Mice were given daily gavage (i.g.) or injected through tail vein every other day (i.v.) for 14 days. (A) For the tumor volume change profiles for each group (was a positive group (i.v.)). (B) For the actual photo of tumor tissues for each group collected after the experiment. (C) For the tumor weight change of mice during the experiment. (D) For the inhibition rate calculated for each group and their statistical analysis. (E) For the mice body weight change profile. (F) For the photo of the fluorescence intensity in tumor mand major organs observed at the 12th h after injection of Dir labeled HICT NRs. (G) For the liver index of each group. (H) For the spleen index of each groups. Data represented as the mean ± SD (*n* = 6); *p < .05 vs. HICT NRs (i.g.).

Antitumor treatment was performed on MCF-7 tumor-bearing nude mice model using PTX injection as a positive control (Fujioka et al., 2017; Zheng et al., 2017). As seen in Figure 5(A, B), daily oral administration of free HICT (35 mg/kg) showed no antitumor effect with a similar tumor growth profile to that of normal saline group, probably due to its poor solubility and oral bioavailability of HICT suspensions even in the presence of TPGS and SO. After fabrication into NRs, HICT displayed a significant tumor inhibitory effect with much slower tumor growth and a much higher tumor inhibition rate (TIR, 47.8%, *p* < .001). When intravenously injected (Figure 5(A)–(D)), HICT NRs dosed every 2 days further demonstrated significantly slower tumor growth and significantly lower TIR (70.4%) than that of daily oral administration (*p* < .05) even dosed every 2 days, close to that for 8 mg/kg of PTX injections (74.5%, TIR), probably due to the high bioavailability and EPR effect.

Dir, a widely used near-red fluorescent dye, was incorporated into HICT NRs and intravenously injected to mice for the last dose to examine the biodistribution of HICT NRs. As shown in Figure 5(F), it was clear that the injected HICT NRs mainly accumulated in liver and tumor, followed by in spleen, with much higher fluorescence than in other organs, demonstrating good tumor targetability.

Although the high biodistribution of HICT NRs in the liver and spleen, there was no difference in the liver index and spleen index between saline group and HICT NRs (i.v.) group (Figure 5(G, H)), suggesting good biosafety of HICT NRs. The similar body weight growth profiles between normal saline group and HICT NRs groups (i.g. and i.v.) (Figure 5(E)) also indicated HICT NRs did not bring mice significant toxicity whatever orally or intravenously administrated.

The good therapeutic efficacy for breast cancer, accumulation in tumor tissue, preliminary good safety, together with potential anti-depression activity, demonstrated that HICT NRs could be a promising antitumor agent for the treatment of breast cancer in clinic.

## Conclusions

Flavonoid compound with presentable antitumor activity is attracting more and more attention, as their various concomitant biological activities beneficial to human health, which may significantly improve the living quality of tumor patients and help them restore their physical power. In our preliminary screening, HICT displayed good growth inhibition against a number of tumor cell lines and immune-enhancing effects (Pan et al., 2007, 2010; Lai et al., 2013; Zhang et al., 2018), but the poor solubility restricted its *in vivo* drug delivery. In this work, we encapsulated HICT into nanoparticles with high DLC and optimize the formulation to achieve good stability in storage and in various physiological medium, thus suitable to oral or intravenous administration. TEM observation that the obtained HICT nanoparticles were NRs in shape, which was never seen before. TEM observation verified that the obtained HICT nanoparticles were NRs in shape, and this is the first NRs report for the nanoparticles of flavonoid compounds. Through the incorporation of small amount of SO, the obtained HICT NRs could be directly lyophilized and then go back to the NR colloidal system of similar size. NR significantly enhanced the cytotoxicity of HICT against MCF-7 breast cancer cells, enhanced the *in vivo* inhibition rate from nearly 0 to 47.8% when orally dosed. Intravenously injection of HICT NRs mainly accumulated liver and tumor, and even achieved similar antitumor therapeutic efficacy to that of PTX injections. It seemed that HICT NRs are promising to be a novel antitumor agent along with much health-helpful effect that may benefit tumor patients in clinic. However, further study and more work are needed to provide more evidence.

## Supplementary Material

Supplemental Material

## References

[CIT0001] Akram M, Iqbal M, Daniyal M, et al. (2017). Awareness and current knowledge of breast cancer. Biol Res 50:33–55. 28969709 10.1186/s40659-017-0140-9 PMC5625777

[CIT0002] Allen C, Maysinger D, Eisenberg A. (1999). Nano-engineering block copolymer aggregates for drug delivery. Colloids Surfaces B: Biointerfaces 16:3–27.

[CIT0003] Banerjee A, Qi J, Gogoi R, et al. (2016). Role of nanoparticle size, shape and surface chemistry in oral drug delivery. J Control Release 238:176–85. 27480450 10.1016/j.jconrel.2016.07.051 PMC5289391

[CIT0004] Chatterjee N, Bivona TG. (2019). Polytherapy and targeted cancer drug resistance. Trends Cancer 5:170–82. 30898264 10.1016/j.trecan.2019.02.003 PMC6446041

[CIT0005] Chen W, Sun K, Zheng R, et al. (2018). Cancer incidence and mortality in China, 2014. Chin J Cancer Res 30:1–12. 29545714 10.21147/j.issn.1000-9604.2018.01.01 PMC5842223

[CIT0006] Dong D, Quan E, Yuan X, et al. (2017). Sodium oleate-based nanoemulsion enhances oral absorption of chrysin through inhibition of UGT-mediated metabolism. Mol Pharmaceutics 14:2864–74. 10.1021/acs.molpharmaceut.6b00851 27983856

[CIT0007] Fujioka H, Sakai A, Tanaka S, et al. (2017). Comparative proteomic analysis of paclitaxel resistance-related proteins in human breast cancer cell lines. Oncol Lett 13:289–95. 28123557 10.3892/ol.2016.5455 PMC5245125

[CIT0008] Guo R, Guo X, Hu X, et al. (2017). Fabrication and optimization of self-microemulsions to improve the oral bioavailability of total flavones of *Hippophae rhamnoides L*. J Food Sci 82:2901–9. 29058766 10.1111/1750-3841.13944

[CIT0009] Guo Y, Zhang X, Meng J, et al. (2011). An anticancer agent icaritin induces sustained activation of the extracellular signal-regulated kinase (ERK) pathway and inhibits growth of breast cancer cells. Eur J Pharmacol 658:114–22. 21376032 10.1016/j.ejphar.2011.02.005 PMC3071461

[CIT0010] Hashizume H, Baluk P, Morikawa S, et al. (2000). Openings between defective endothelial cells explain tumor vessel leakiness. Am J Pathol 156:1363–80. 10751361 10.1016/S0002-9440(10)65006-7 PMC1876882

[CIT0011] He J, Shi H, Huang S, et al. (2018). Core-shell nanoencapsulation of α-tocopherol by blending sodium oleate and rebaudioside A: preparation, characterization, and antioxidant activity. Molecules 23:3183–93. 10.3390/molecules23123183 PMC6321206 30513920

[CIT0012] Lai X, Ye Y, Sun C, et al. (2013). Icaritin exhibits anti-inflammatory effects in the mouse peritoneal macrophages and peritonitis model. Int Immunopharmacol 16:41–9. 23566810 10.1016/j.intimp.2013.03.025

[CIT0013] Lewin G, Maciuk A, Moncomble A, et al. (2013). Enhancement of the water solubility of flavone glycosides by disruption of molecular planarity of the aglycone moiety. J Nat Prod 76:8–12. 23249276 10.1021/np300460a

[CIT0014] Maeda H, Nakamura H, Fang J. (2013). The EPR effect for macromolecular drug delivery to solid tumors: Improvement of tumor uptake, lowering of systemic toxicity, and distinct tumor imaging in vivo. Adv Drug Deliv Rev 65:71–9. 23088862 10.1016/j.addr.2012.10.002

[CIT0015] Pan Y, Kong LD, Li YC, et al. (2007). Icariin from Epimedium brevicornum attenuates chronic mild stress-induced behavioral and neuroendocrinological alterations in male Wistar rats. Pharmacol Biochem Behav 87:130–40. 17509675 10.1016/j.pbb.2007.04.009

[CIT0016] Pan Y, Wang FM, Qiang LQ, et al. (2010). Icariin attenuates chronic mild stress-induced dysregulation of the LHPA stress circuit in rats. Psychoneuroendocrinology 35:272–83. 19631474 10.1016/j.psyneuen.2009.06.020

[CIT0017] Peng J, Sengupta S, Jordan VC. (2009). Potential of selective estrogen receptor modulators as treatments and preventives of breast cancer. ACAMC 9:481–99. 10.2174/187152009788451833 PMC3767174 19519291

[CIT0018] Perez-Vizcaino F, Fraga CG. (2018). Research trends in flavonoids and health. Arch Biochem Biophys 646:107–12. 29580946 10.1016/j.abb.2018.03.022

[CIT0019] Prabhakar U, Maeda H, Jain RK, et al. (2013). Challenges and key considerations of the enhanced permeability and retention effect for nanomedicine drug delivery in oncology. Cancer Res 73:2412–7. 23423979 10.1158/0008-5472.CAN-12-4561 PMC3916009

[CIT0020] Rao WW, Yang MJ, Cao BN, et al. (2019). Psychological distress in cancer patients in a large Chinese cross-sectional study. J Affective Disord 245:950–6. 10.1016/j.jad.2018.11.089 30699880

[CIT0021] Rauf A, Imran M, Khan IA, et al. (2018). Anticancer potential of quercetin: a comprehensive review. Phytother Res 32:2109–30. 30039547 10.1002/ptr.6155

[CIT0022] Shah R, Rosso K, Nathanson SD. (2014). Pathogenesis, prevention, diagnosis and treatment of breast cancer. World J Clin Oncol 5:283–98. 25114845 10.5306/wjco.v5.i3.283 PMC4127601

[CIT0023] Stanton AL, Bower JE. (2015). Psychological adjustment in breast cancer survivors. Adv Exp Med Biol 862:231–42. 26059939 10.1007/978-3-319-16366-6_15

[CIT0024] Torchilin V. (2011). Tumor delivery of macromolecular drugs based on the EPR effect. Adv Drug Delivery Rev 63:131–5. 10.1016/j.addr.2010.03.011 20304019

[CIT0025] Torre LA, Bray F, Siegel RL, et al. (2015). Global cancer statistics. CA Cancer J Clin 65:87–108. 25651787 10.3322/caac.21262

[CIT0026] Toy R, Peiris PM, Ghaghada KB, et al. (2014). Shaping cancer nanomedicine: the effect of particle shape on the in vivo journey of nanoparticles. Nanomedicine 9:121–34. 24354814 10.2217/nnm.13.191 PMC4057606

[CIT0027] Wang X, Zheng N, Dong J, et al. (2017). Estrogen receptor-alpha36 is involved in icaritin induced growth inhibition of triple-negative breast cancer cells. J Steroid Biochem Mol Biol 171:318–27. 28529129 10.1016/j.jsbmb.2017.05.009

[CIT0028] Yang H, Chen Z, Zhang L, et al. (2016). Mechanism for the Cellular Uptake of Targeted Gold Nanorods of Defined Aspect Ratios. Small 12:5178–89. 27442290 10.1002/smll.201601483

[CIT0029] Yang L, Alexandridis P. (2000). Physicochemical aspects of drug delivery and release from polymer-based colloids. Curr Opin Colloid Interface Sci 5:132–43.

[CIT0030] Zainal NZ, Nik-Jaafar NR, Baharudin A, et al. (2013). Prevalence of depression in breast cancer survivors: a systematic review of observational studies. Asian Pac J Cancer Prev 14:2649–56. 23725190 10.7314/apjcp.2013.14.4.2649

[CIT0031] Zhang K, Dai Z, Liu R, et al. (2018). Icaritin provokes serum thrombopoietin and downregulates thrombopoietin/MPL of the bone marrow in a mouse model of immune thrombocytopenia. Mediators Inflammation 8:7235639–48. 10.1155/2018/7235639 PMC6129856 30224899

[CIT0032] Zheng X, Wang C, Xing Y, et al. (2017). SB-T-121205, a next-generation taxane, enhances apoptosis and inhibits migration/invasion in MCF-7/PTX cells. Int J Oncol 50:893–902. 28197640 10.3892/ijo.2017.3871 PMC5358697

