# Supplementary material for: Hydrous icaritin nanorods with excellent stability improves the in vitro and in vivo activity against breast cancer
Source: Drug Deliv. 2020 Jan 31;27(1):228–37. doi: 10.1080/10717544.2020.1716877 (PMC7034031; doi:10.1080/10717544.2020.1716877)
Supplement: Supplemental Material [file IDRD_A_1716877_SM0988.docx]

**Table S1**. Particle size, PDI and zeta potential of HICT nanosuspensions with PCL_2k_-mPEG_2k_ as the carrier after 10 hours of incubation in various physiological medium. (mean ± SD).

| **Medium** | **Size（nm）** | **PDI** | **Zeta（mV）** |
| --- | --- | --- | --- |
| **PBS** | 5903±527.5 | 0.489±0.028 | -2.45±0.45 |
| **5 % Glu** | 4919±864.8 | 0.501±0.017 | -1.01±0.565 |
| **0.9 % NaCl** | 263.2±3.0 | 0.23±0.04 | -8.43±0.3 |
| [**Gastric**](javascript:;) [**fluid**](javascript:;) | 9093±407.3 | 0.299±0.047 | -3.56±1.0 |
| [**Intestinal**](javascript:;) [**fluid**](javascript:;) | 8420±545.2 | 0.301±0.046 | -2.8±1.05 |

**Table S2.** Particle size, PDI and zeta potential of HICT nanosuspensions with PLA_2k_-mPEG_2k_ as the carrier after 10 hours of incubation in various physiological medium. (mean ± SD).

| **Medium** | **Size（nm）** | **PDI** | **Zeta（mV）** |
| --- | --- | --- | --- |
| **PBS** | 1.65×10^4^±128.0 | 1.0±0 | -10.3±0.33 |
| **5 % Glu** | 1.8×10^4^±236.0 | 0.8±0.2 | -11±0.23 |
| **0.9 % NaCl** | 265.3±4.6 | 0.28±0.02 | -6.15±0.1 |
| [**Gastric**](javascript:;) [**fluid**](javascript:;) | 3103±56.4 | 0.127±0.04 | 5.80±0.5 |
| [**Intestinal**](javascript:;) [**fluid**](javascript:;) | 5658±102.3 | 0.319±0.012 | -14.5±0.45 |

**Table S3**. Particle size, PDI and zeta potential of HICT nanosuspensions with sodium oleate as the carrier after 10 hours of incubation in various physiological medium. (mean ± SD).

| **Medium** | **Size（nm）** | **PDI** | **Zeta（mV）** |
| --- | --- | --- | --- |
| **PBS** | 1.1×10^4^±2255 | 0.709±0.313 | -12.4±1.06 |
| **5 % Glu** | 2.1×10^4^±1242 | 0.463±0.223 | -0.02±0.09 |
| **0.9 % NaCl** | 279.4±3.4 | 0.32±0.05 | -70.5±1.0 |
| [**Gastric**](javascript:;) [**fluid**](javascript:;) | 4398±755 | 0.577±0.149 | 7.90±0.189 |
| [**Intestinal**](javascript:;) [**fluid**](javascript:;) | 2594±222.1 | 0.417±0.358 | -10.1±0.07 |

**Table S4**. Particle size, PDI and zeta potential of HICT nanosuspensions with TPGS as the carrier after 10 hours of incubation in various physiological medium. (mean ± SD)

| **Medium** | **Size（nm）** | **PDI** | **Zeta（mV）** |
| --- | --- | --- | --- |
| **PBS** | 1602±24.04 | 0.458±0.049 | -0.426±013 |
| **5 % Glu** | 984.5±7.03 | 0.448±0.054 | -0.99±0.4 |
| **0.9 % NaCl** | 225.6±6.7 | 0.20±0.02 | -14.4±0.06 |
| [**Gastric**](javascript:;) [**fluid**](javascript:;) | 1170±24.02 | 0.197±0.056 | 4.80±0.24 |
| [**Intestinal**](javascript:;) [**fluid**](javascript:;) | 657.6±4.74 | 0.277±0.058 | -1.69±0.3 |

**Table S5**. Particle size, PDI and zeta potential of HICT nanosuspensions with SPC as the carrier after 10 hours of incubation in various physiological medium. (mean ± SD)

| **Medium** | **Size（nm）** | **PDI** | **Zeta（mV）** |
| --- | --- | --- | --- |
| **PBS** | 1111±22.68 | 0.218±0.092 | -0.730±0.12 |
| **5 % Glu** | 1408±47.26 | 0.271±0.085 | -1.6±0.71 |
| **0.9 % NaCl** | 445.2±37 | 0.46±0.06 | -12.9±0.2 |
| [**Gastric**](javascript:;) [**fluid**](javascript:;) | 1903±76.74 | 0.216±0.072 | -1.1±0.23 |
| [**Intestinal**](javascript:;) [**fluid**](javascript:;) | 1417±100.3 | 0.248±0.066 | -1.77±0.56 |

**Table S6**. Particle size, PDI and zeta potential of HICT nanosuspensions with BSA as the carrier after 10 hours of incubation in various physiological medium. (mean ± SD)

| **Medium** | **Size（nm）** | **PDI** | **Zeta（mV）** |
| --- | --- | --- | --- |
| **PBS** | 1980±215.1 | 0.440±0.357 | -2.82±1.41 |
| **5 % Glu** | 832.8±109.7 | 0.503±0.053 | 6.99±1.2 |
| **0.9 % NaCl** | 392±11.0 | 0.38±0.04 | -10.1±0.2 |
| [**Gastric**](javascript:;) [**fluid**](javascript:;) | 8256±3110 | 0.517±0.154 | -7.3±0.98 |
| [**Intestinal**](javascript:;) [**fluid**](javascript:;) | 6046±59.9 | 0.4±0.09 | -4.16±0.45 |

**Table S7**. Particle size, PDI and zeta potential of HICT nanosuspensions with the HICT/TPGS feeding ratio of 2:1 after 10 hours of incubation in various physiological medium. (mean ± SD)

| **Medium** | **Size（nm）** | **PDI** | **Zeta（mV）** |
| --- | --- | --- | --- |
| [**Initial**](javascript:;) | 316.0±4.7 | 0.113±0.02 | -27.0±0.05 |
| **PBS** | 362.1±5.4 | 0.263±0.03 | -3.51±0.2 |
| **5%Glu** | 382.3±3.0 | 0.150±0.02 | -10.5±0.8 |
| **0.9%NaCl** | 338.5±3.8 | 0.169±0.05 | -2.37±0.7 |
| [**Gastric**](javascript:;) [**fluid**](javascript:;) | 361.7±5.6 | 0.270±0.04 | 3.22±0.2 |
| [**Intestinal**](javascript:;) [**fluid**](javascript:;) | 363.3±6.7 | 0.248±0.02 | -2.13±0.01 |

**Table S8**. Particle size, PDI and zeta potential of HICT nanosuspensions with the HICT/TPGS feeding ratio of 3:1 after 10 hours of incubation in various physiological medium. (mean ± SD)

| **Medium** | **Size（nm）** | **PDI** | **Zeta（mV）** |
| --- | --- | --- | --- |
| [**Initial**](javascript:;) | 209.8±4.7 | 0.151±0.047 | -34.4.5±1.5 |
| **PBS** | 257.1±6.6 | 0.203±0.02 | -2.67±0.3 |
| **5%Glu** | 279.0±4.0 | 0.184±0.01 | -16.4±0.9 |
| **0.9%NaCl** | 246.4±5.2 | 0.127±0.02 | -8.47±0.4 |
| [**Gastric**](javascript:;) [**fluid**](javascript:;) | 279.8±2.5 | 0.159±0.01 | -1.43±0.1 |
| [**Intestinal**](javascript:;) [**fluid**](javascript:;) | 261.5±3.5 | 0.239±0.03 | -2.35±0.1 |

**Table S9**. Particle size, PDI and zeta potential of HICT nanosuspensions with the HICT/TPGS feeding ratio of 4:1 after 10 hours of incubation in various physiological medium. (mean ± SD)

| **Medium** | **Size（nm）** | **PDI** | **Zeta（mV）** |
| --- | --- | --- | --- |
| [**Initial**](javascript:;) | 351.1±6.3 | 0.192±0.02 | -17.6±1.9 |
| **PBS** | 433.9±6.5 | 0.198±0.01 | -3.23±0.7 |
| **5%Glu** | 406.6±4.7 | 0.095±0.03 | -8.12±0.5 |
| **0.9%NaCl** | 407.5±8.1 | 0.199±0.04 | -3.09±0.9 |
| [**Gastric**](javascript:;) [**fluid**](javascript:;) | 459.2±10.1 | 0.273±0.09 | -4.68±0.2 |
| [**Intestinal**](javascript:;) [**fluid**](javascript:;) | 430.7±7.3 | 0.254±0.03 | 1.78±0.9 |

**Table S10.** The values of different experiment groups against MCF-7 tumor in vivo (mean ± SD)

| **Prescription** | **Tumor weight (g)** | **Inhibition rate (%)** |
| --- | --- | --- |
| Saline (i.v) | 1.74±0.34 |  |
| Free HICT (i.g) | 1.78±0.83 |  |
| HICT NRs (i.g) | 0.91±0.36 | 47.8±17.2 |
| HICT NRs (i.v) | 0.51±0.24 | 70.4±12.9* |
| PTX injection (i.v) | 0.44±0.11 | 74.5±6.7* |

*P<0.05 vs HICT NRs (i.g)

**Table S11**.The values of different groups liver and spleen index. (mean ± SD)

| **Group** | **Liver index** | **Spleen index** |
| --- | --- | --- |
| Saline (i.v) | 0.05±0.010 | 0.0069±0.0022 |
| Free HICT (i.g) | 0.056±0.0046 | 0.0083±0.0019 |
| HICT NRs (i.v) | 0.057±0.013 | 0.0058±0.0012 |
| HICT NRs (i.g) | 0.051±0.005 | 0.0063±0.0019 |
| PTX injection (i.v) | 0.047±0.004 | 0.0055±0.0008 |
